# Ag-Modified Porous Perovskite-Type LaFeO_3_ for Efficient Ethanol Detection

**DOI:** 10.3390/nano12101768

**Published:** 2022-05-22

**Authors:** Jiejie Yu, Cong Wang, Quan Yuan, Xin Yu, Ding Wang, Yang Chen

**Affiliations:** 1 School of Materials and Chemistry, University of Shanghai for Science & Technology, Shanghai 200093, China; m15370026136@163.com (J.Y.); wangcong_0311@163.com (C.W.); yquan0327@163.com (Q.Y.); yuxin156625861@163.com (X.Y.); 2 NEST Lab, Department of Physics, Department of Chemistry, College of Sciences, Shanghai University, Shanghai 200444, China; 3 Shanghai Yaolu Instrument & Equipment Co., Ltd., Shanghai 200444, China

**Keywords:** perovskite, Ag/LaFeO_3_, ethanol sensing, gas sensor

## Abstract

Perovskite (ABO_3_) nanosheets with a high carrier mobility have been regarded as the best candidates for gas-sensitive materials arising from their exceptional crystal structure and physical–chemical properties that often exhibit good gas reactivity and stability. Herein, Ag in situ modified porous LaFeO_3_ nanosheets were synthesized by the simple and efficient graphene oxide (GO)-assisted co-precipitation method which was used for sensitive and selective ethanol detection. The Ag modification ratio was studied, and the best performance was obtained with 5% Ag modification. The Ag/LaFeO_3_ nanomaterials with high surface areas achieved a sensing response value (R_g_/R_a_) of 20.9 to 20 ppm ethanol at 180 °C with relatively fast response/recovery times (26/27 s). In addition, they showed significantly high selectivity for ethanol but only a slight response to other interfering gases. The enhanced gas-sensing performance was attributed to the combination of well-designed porous nanomaterials with noble metal sensitization. The new approach is provided for this strategy for the potential application of more P-type ABO_3_ perovskite-based gas-sensitive devices.

## 1. Introduction

ABO_3_ perovskite-type oxides have been widely applied in various fields, such as catalysts [1,2], Li-O_2_ batteries [3,4], magnetic materials [5] and gas sensors [6,7,8]. ABO_3_ consists of a metal cation with a strong thermal stability at the large ionic radius A-site and a B-O octahedral structure providing the active site. Its positive crystal structure and physical–chemical properties tend to give superior gas reactivity and stability, which offer huge potential for the detection of VOCs [9,10]. As a classical ABO_3_ material, LaFeO_3_ has superior p-type electron conductivity, outstanding oxygen ion mobility and catalytic activity rendering it a suitable candidate for gas-sensing applications [11,12,13,14]. However, it still has a few difficulties to overcome in order to suit practical requirements, such as a poor gas sensitivity and higher operating temperatures. Theoretically, the response value of a p-type MOS would be the square root of an n-type MOS if they have the same dimensions and morphology [15]. In particular, a high operating temperature increases electrical consumption and reduces gas sensitivity caused by the desorption of target gases and surface oxygen ions [16]. Nevertheless, broadening the gas-sensitive properties of LaFeO_3_ perovskite materials is of great importance.

Operating temperature, sensitivity, response/recovery time, selectivity and stability are all critical factors in the application of semiconductor gas sensor materials, which in turn are closely tied to the morphology, size, surface area to volume ratio, modification and so on [17,18]. Porous nanosheets have demonstrated excellent physical, chemical, mechanical, electronic and surface properties that hold promise and appeal [19,20]. In recent years, porous sensitive nanomaterials have increasingly been used owing to their higher surface area to volume ratio, active surface oxygen vacancy concentration and excellent oxygen storing capacity [21,22]. To date, the graphene oxide (GO) template method has been adopted for the preparation of various single metal oxide nanosheets, such as ZnO [23], TiO_2_ [24], SnO_2_ [25], Co_3_O_4_ [26], as well as others. Nevertheless, few reports have been devoted to the preparation of porous perovskite-type polymetallic oxide nanomaterials. In addition, noble metal modifications are common means of reducing resistance and increasing sensitivity. Notably, Ag modification is an appealing approach to achieve a better sensing performance on account of its excellent chemically and electronically sensitive catalytic properties [27,28]. With the sensitized atoms also generating further adsorption sites for atmospheric oxygen, the target gas molecules contribute meaningfully to the exchange on the base surface and the adsorbate [29]. Therefore, well-designed, non-toxic, porous nanomaterials combined with active noble metal sensitization is a highly promising approach.

In this work, ultra-thin, porous, Ag-modified LaFeO_3_ nanosheets with perovskite structures were successfully synthesized via the graphene oxide (GO) template method and subsequent thermal treatment. The effect of the Ag-modified content on the morphology, structure and ethanol gas-sensitive performance of the product was also investigated. In addition, an exploration of the mechanism of Ag modification on its gas-sensitive performance enhancement was carried out. Finally, a possible mechanism for the enhanced gas-sensitive performance of Ag/LaFeO_3_ was explored.

## 2. Materials and Methods

### 2.1. Materials

Lanthanum(III) nitrate hexahydrate (La(NO_3_)_3_·6H_2_O), ferric nitrate nonahydrate (Fe(NO_3_)_3_·9H_2_O), sodium hydroxide (NaOH, 98%), ethylene glycol (EG, 99%) and ethanol (EtOH, 99.7%) were purchased from Sigma-Aldrich (Burlington, MA, USA). Graphene oxide (GO) was purchased from Changzhou Sixth Element Material Technology Co., Ltd. (Changzhou, China). All chemical reagents were available in analytical grade and suitable for use without further purification.

### 2.2. Methods

Synthesis of ultrathin porous LaFeO_3_ nanosheets: As part of the typical procedure, GO (80 mg) was added into 168 mL ethylene glycol with 32 mL deionized water to form a clear solution with an ultrasound for 30 min. Then, 21.6 mg La(NO_3_)_3_·6H_2_O and 20.2 mg Fe(NO_3_)_3_·9H_2_O were added into the ethylene glycol solution followed by the addition of 140 mg NaOH with an ultrasound for around 30 min. Then, the mixture was stirred at room temperature for 4 h. Finally, the precipitate was collected, washed with ethanol and deionized water three times and freeze-dried for 24 h. After drying, the powder was calcined in air at a heating rate of 2 °C/min and kept at a certain temperature for 2 h to obtain the ultrathin porous LaFeO_3_ nanosheets.

Synthesis of ultrathin porous Ag/LaFeO_3_ nanosheets: Ag/LaFeO_3_ nanosheets were synthesized via a similar procedure to the synthesis of LaFeO_3_ nanosheets except that a certain amount of AgNO_3_ solution was added in the former before centrifugation. The Ag/La ratios of the prepared powers were 0, 0.01, 0.05 and 0.10. The as-generated Ag-modified LaFeO_3_ nanosheets are herein denoted as Ag/LaFeO_3_-0, Ag/LaFeO_3_-1, Ag/LaFeO_3_-5 and Ag/LaFeO_3_-10, respectively.

### 2.3. Characterization

LaFeO_3_ nanosheets and Ag/LaFeO_3_ nanosheets were analyzed via the following characterization methods. The morphology of the samples was visualized by FE-SEM (FEI, Quanta FEG 450, Houston, TX, USA). Transmission electron microscope (TEM) images and high-resolution transmission electron microscope (HRTEM) images were accessed by a Tecnai G220S-Twin transmission electron microscope operated at 120 and 200 kV accelerating voltages to explore the morphology and microstructure of the samples. Prior to measurement, samples were sonicated and dispersed in ethanol, then immersed in a copper grid with a lacey carbon film and dried at room temperature. An X-ray diffractometer (Bruker, D8 Advance, Bremen, Germany) with Cu-Kα (λ = 0.15418 nm) recorded the phases of the solid powders. Moreover, the XRD patterns were collected over a 2θ range of 20–70° in 5° min^−1^ steps at room temperature. The chemical state of the surface of the samples was appraised by X-ray photoelectron spectroscopy (XPS, Thermo Scientific, Waltham, MA, USA, ESCALAB 250) with Mg Kα radiation. The C1s photoelectron peak (284.6 eV) was used as a reference to calibrate the binding energy. TG/DSC was measured from 25 to 1000 °C in the air at a heating rate of 5 °C/min with a thermal analyzer (TG, STA449C). Brunauer–Emmett–Teller (BET) surface area and pore size distribution investigations were carried out on an ASIC-2 gas adsorption analyzer. (N_2_ as the adsorbate and operation temperature = −196 °C).

### 2.4. Gas Sensor Fabrication and Measurement

The samples were mixed with deionized water at a certain ratio and grated into a paste which was applied to the ceramic tube elements where a thin sensing film was formed. Each end of the ceramic tube was fitted with a pair of gold electrodes attached to two platinum wires on each electrode. The ceramic tubes were inserted into a Ni-Cr heating wire resulting in an indirectly heated gas sensor (Figure S1). The design and details of the ceramic sensor were as reported previously in the literature [30]. In this paper, the gas-sensing performances of the gas sensors were measured with the commercial CGS-8 gas-sensing measurement system by recording the change of resistance of the gas sensors.

## 3. Results and Discussion

### 3.1. Characterization of the Sensing Materials

**Scheme 1:**
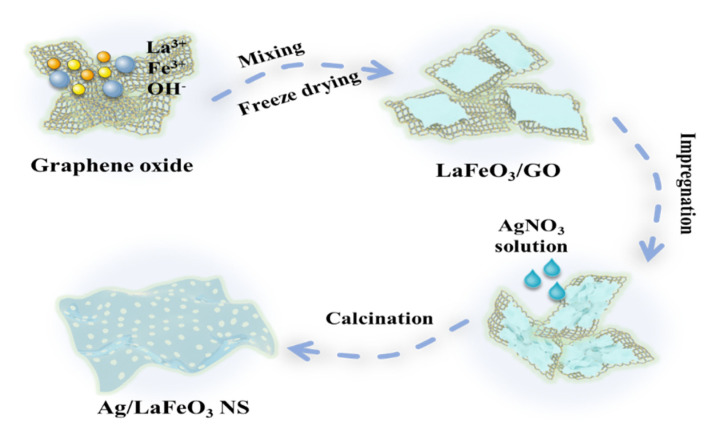
The schematic diagram of the synthesis of Ag/LaFeO_3_ nanosheets by the GO template method.

The synthesis process of the porous Ag/LaFeO_3_ nanosheets is described in Scheme 1. The precursor LaFeO_3_/GO was synthesized by the graphene oxide-assisted co-precipitation method. Subsequently, the precursor was impregnated with different concentrations of AgNO_3_ solution. Then, the Ag/LaFeO_3_ nanosheets were obtained by heat treatment. Usually, the high calcination temperature is prone to sintering, which accounts for the drop in the active specific surface area, while the low temperature does not lead to crystallinity. As a gas-sensitive material, LaFeO_3_ requires both a high specific surface area and a certain degree of crystallinity, which requires the study of the appropriate calcination temperature.

To investigate the thermal behavior of the LaFeO_3_/GO template hybrid precursors, TG tests were carried out under an air atmosphere in the range of 50~1000 °C with a heating rate of 5 °C·min^−1^, as shown in Figure S2a. According to the TG curves, the thermal performance of the LaFeO_3_/GO nanocomposites improved significantly with the rise in temperature. The first loss of weight until 110 °C (5.4%) was mainly responsible for the removal of adsorbed water from the sample. The second stage of weight loss (16.8%) could be attributed to the loss of ethylene glycol and the degradation of the residual organic functional groups remaining on the graphene oxide template. The weight loss in the third stage (19.7%) was due to the combustion of the residual nitrates. The weight loss in the fourth stage (38%) was recognized as the combustion of graphene oxide [31]. Therefore, it was reasonable to choose the calcination temperature of 500–800 °C.

**Figure 1:**
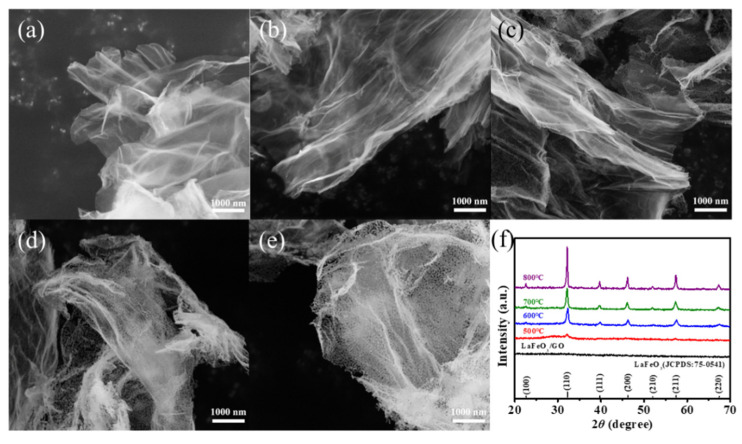
SEM micrographs of LaFeO_3_ at different calcination temperatures. (**a**) LaFeO_3_/GO; (**b**) LaFeO_3_/GO calcined at 500 °C; (**c**) LaFeO_3_/GO calcined at 600 °C; (**d**) LaFeO_3_/GO calcined at 700 °C; (**e**) LaFeO_3_/GO calcined at 800 °C; (**f**) XRD patterns of the samples.

The morphology of LaFeO_3_ calcined at different temperatures was characterized. Figure 1a–e are the SEM images of LaFeO_3_/GO and LaFeO_3_ obtained after the calcination of LaFeO_3_/GO at 500–800 °C, respectively. All these products were ultrathin porous nanosheets with rolled-up edges due to the surface tension, which was caused by the GO template. It was found that the LaFeO_3_/GO intact ultrathin nanosheets gradually turned into porous nanosheets composed of particles with the increase in calcination temperature. In Figure 1d–e, the porous nanosheets assembled by nanoparticles can be clearly observed. In addition, the pore size of the ultrathin nanosheets increased due to the gradual increase in nanoparticles.

The XRD patterns of the LaFeO_3_/GO and LaFeO_3_ materials are given in Figure 1f. Among them, the nanosheets of LaFeO_3_/GO showed an amorphous structure. Correspondingly, in the Raman spectrum in Figure S2b, the peaks of the curves corresponding to LaFeO_3_/GO at around 1347 cm^−1^ and 1588 cm^−1^ could be attributed to the D and G bands of the graphene plane [32]. In addition, the diffraction peaks of LaFeO_3_ calcined at four different temperatures matched well with the standard LaFeO_3_ (JCPDS: 75-0541) perovskite structure [33]. This implies that all the prepared LaFeO_3_ sensing materials had a perovskite crystal structure with orthorhombic phases when calcined at 500 °C and higher. The XRD patterns showed that the crystallinity of the prepared LaFeO_3_ nanosheets increased significantly with the increase in calcination temperature. The working temperature had a significant effect on the oxygen adsorbed on the sensing materials surface, and it was a major factor affecting the gas-sensitive performance.

**Figure 2:**
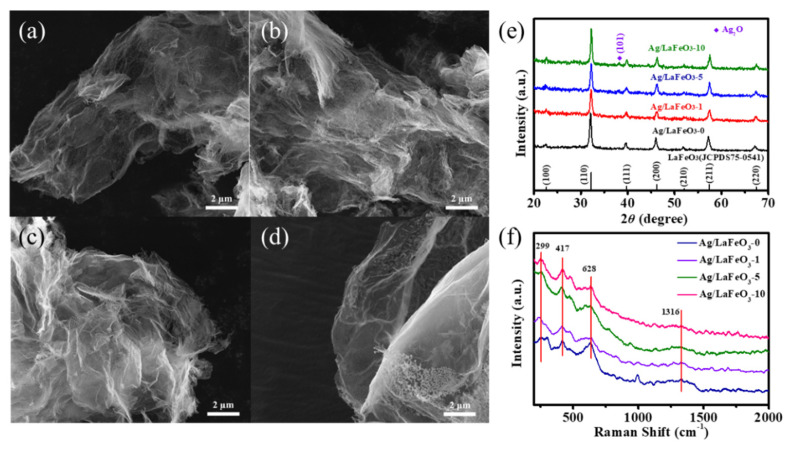
SEM images of (**a**) Ag/LaFeO_3_-0; (**b**) Ag/LaFeO_3_-1; (**c**) Ag/LaFeO_3_-5; (**d**) Ag/LaFeO_3_-10; (**e**) XRD patterns of the Ag/LaFeO_3_ samples with different calcination temperatures; (**f**) Raman spectra of the Ag/LaFeO_3_ samples.

The morphologies of Ag/LaFeO_3_ nanosheets (Ag/LaFeO_3_-0, Ag/LaFeO_3_-1, Ag/LaFeO_3_-5, and Ag/LaFeO_3_-10) were characterized by SEM. As shown in Figure 2a, the layer sizes of the Ag/LaFeO_3_-0 nanosheets were approximately 5 μm after calcination at 700 °C, which was determined by the size of the sacrificial GO template. Figure 2b–d shows the SEM patterns of Ag/LaFeO_3_-1, Ag/LaFeO_3_-5 and Ag/LaFeO_3_-10, respectively. There were nano rod-like shadows attached to the nanosheets, which tended to grow with Ag modifications. The XRD patterns provided information on the crystallinity and the perovskite phase of the samples. As shown in Figure 2e, all four prepared samples of Ag-modified ultrathin LaFeO_3_ nanosheets showed a distinct LaFeO_3_ perovskite phase, and the main peaks appearing in the plots corresponded to the diffraction of (100), (110), (200), (210), (211) and (221) crystal planes, which matched perfectly with the standard JCPDS card 75-0541 [33]. Only weak Ag_2_O (JCPDS: 19-1155) diffraction peaks were detected in the Ag/LaFeO_3_-10 sample corresponding to the peak of (101) crystal plane diffraction due to the low Ag content and high dispersion of the samples [29]. The Ag/LaFeO_3_ samples were further studied by Raman spectroscopy. As illustrated in Figure 2f, four Raman peaks at 299, 417, 628 and 1316 cm^−1^ mapped to the orthogonal LaFeO_3_ structure [31]. The 299 cm^−1^ (Ag mode) peak was correlated with La-O, and the 417 cm^−1^ peak denoted the in-plane Fe-O (B_3g_) vibrational mode. Moreover, the 628 and 1316 cm^−1^ peaks were associated with the two-photon scattering of O^2−^ [34].

The response of the LaFeO_3_ samples to 50 ppm ethanol at an operating temperature of 140–220 °C is shown in Figure S3. From the response curves, it is clear that LaFeO_3_ calcined at 700 °C had the best response at 180 °C. Therefore, the calcination temperature was chosen to be 700 °C for the subsequent material study. In addition, the gas-sensitive properties of Ag/LaFeO_3_-5 materials were better than those of Ag/LaFeO_3_-0, Ag/LaFeO_3_-1 and Ag/LaFeO_3_-10. Therefore, the following discussions will focus on the Ag/LaFeO_3_-5 material.

**Figure 3:**
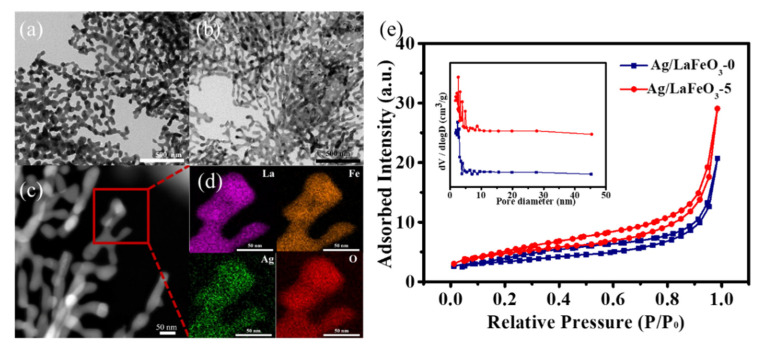
TEM of (**a**) Ag/LaFeO_3_-0; and (**b**) Ag/LaFeO_3_-5; (**c**,**d**) the STEM-EDS elemental mapping images of Ag/LaFeO_3_-5; (**e**) BET characterization of Ag/LaFeO_3_-0 and Ag/LaFeO_3_-5, and the inset shows the pore size distribution of the corresponding composites.

The porous structure and morphologies of the Ag/LaFeO_3_-0 and Ag/LaFeO_3_-5 samples were further characterized by TEM. The nanosheets were formed by many irregular dendritic nanocrystals. These dendritic nanocrystals were uniformly distributed and the Ag modification did not change their microscopic morphology (Figure 3a,b). To further determine the elemental distribution of these “dendrites”, the STEM-EDS method was used. From Figure 3c,d, it can be clearly observed that the La, Fe, Ag and O elements were well distributed in the three-dimensional space of the Ag/LaFeO_3_-5 sample, indicating that the Ag elements were homogeneously dispersed on the whole LaFeO_3_ sample.

The pore structure and specific surface area of the Ag/LaFeO_3_ ultrathin nanosheets were investigated using nitrogen adsorption and desorption isotherms. A significant hysteresis in the type IV adsorption isotherm at higher relative pressures indicated the presence of mesoporous structures (Figure 3e). The BET specific surface area of Ag/LaFeO_3_-5 (16.45 m^2^/g) was larger than that of Ag/LaFeO_3_-0 (11.31 m^2^/g), indicating that the Ag modification had a positive effect on the porous morphology. The pore diameters of Ag/LaFeO_3_-5 and Ag/LaFeO_3_-0 were calculated based on the BJH method to be about 6.19 nm and 6.44 nm, respectively. The large specific surface area and abundant mesopores allowed sufficient space to promote the accelerated diffusion of gas molecules and thus improve the gas-sensitive performance.

**Figure 4:**
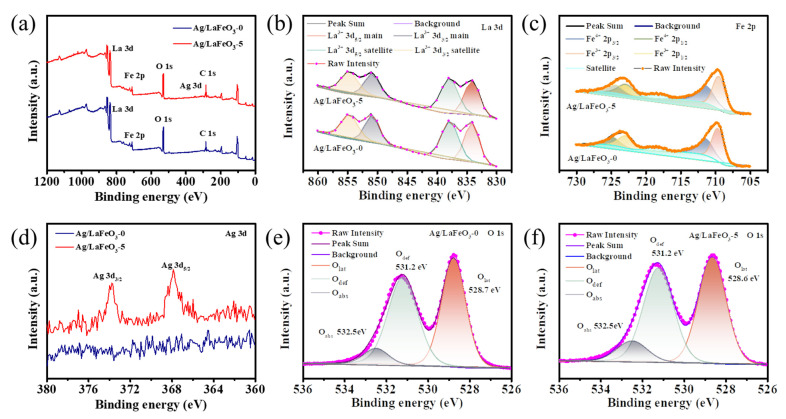
(**a**) XPS survey spectra of Ag/LaFeO_3_-0 and Ag/LaFeO_3_-5, high-resolution core-level XPS spectra of; (**b**) La 3d; (**c**) Fe 2p; (**d**) Ag 3d; (**e**) O 1s of Ag/LaFeO_3_-0; and (**f**) O 1s of Ag/LaFeO_3_-5.

To understand the surface chemical composition and chemical state of the Ag/LaFeO_3_-0 and Ag/LaFeO_3_-5 samples, XPS analysis was used for characterization. The full spectra of the XPS measurement verifying the existence of La, Fe, O and C elements in the Ag/LaFeO_3_-0 and Ag/LaFeO_3_-5 samples are shown in Figure 4a. Among them, Ag elemental was detected in the Ag/LaFeO_3_-5 sample. As shown in Figure 4b, the peaks at 834.1 eV and 850.7 eV corresponded to La 3d_5/2_ and La 3d_3/2_, respectively. The two peaks at 837.8 eV and 854.5 eV corresponded to the La 3d_5/2_ and La 3d_3/2_ satellite peaks, respectively. The typical double peak could be attributed to the splitting of the 3d_5/2_ and 3d_3/2_ spin orbits of the La^3+^ ion, with nuclear holes and electrons, which shifted from the O 2p valence band to the vacant La 4f orbital. The La 3d spectrum indicated that the lanthanide ion was in the La^3+^ valence state [10,11,29]. Further, in the high-resolution Fe 2p spectra (Figure 4c), peaks at 711.4 and 724.9 eV of binding energy could be attached to 2p_3/2_ and 2p_1/2_ of Fe^4+^, respectively, while the 709.6 eV and 722.8 eV peaks could be ascribed to 2p_3/2_ and 2p_1/2_ of Fe^3+^ [10,12,13]. The Ag element (Figure 4d) appeared at binding energies of 367.3 eV and 373.3 eV, respectively, corresponding to the Ag 3d_5/2_ and Ag 3d_3/2_ double peaks of Ag_2_O [35,36,37,38]. This suggests that certain Ag species in the Ag/LaFeO_3_-5 were available in the oxidized state of Ag_2_O. The O 1s spectrum could be an inverse product of three peaks, each corresponding to a different kind of surface chemical state as shown in Figure 4e,f. The binding energy of 528.9 eV was tied to lattice oxygen (O_lat_), the shaded area of 531.2 eV was dedicated to defect oxygen (O_def_), and the surface adsorbed oxygen molecules (O_abs_) counted on a weak peak at 532.4 eV [6]. The lattice oxygen could be attributed to La-O and Fe-O in the LaFeO_3_ lattice. The approximate relative percentage of each oxygen species on the surface of Ag/LaFeO_3_-0 and Ag/LaFeO_3_-5 are summarized in Table S1. The O_lat_ percentage of Ag/LaFeO_3_-0 and Ag/LaFeO_3_-5 were 46.71% and 46.25%, respectively. Furthermore, compared with the O_def_ percentage of the Ag/LaFeO_3_-5 (44.28%), the Ag/LaFeO_3_-0 (46.55%) showed an increased O_def_ percentage. On the contrary, the comparative percentage of O_ads_ for the Ag/LaFeO_3_-5 nanosheets was 9.47%, exhibiting a slightly higher oxygen adsorption percentage than the Ag/LaFeO_3_-0 nanocomposite (6.74%). Based on the above analysis, the Ag/LaFeO_3_-5 could deliver more sorbent oxygen species than Ag/LaFeO_3_-0 nanosheets, which played a significant function in the response with the target gas and could widely enhance the gas-sensing capability of the sensor.

### 3.2. Ethanol-Sensing Performance

**Figure 5:**
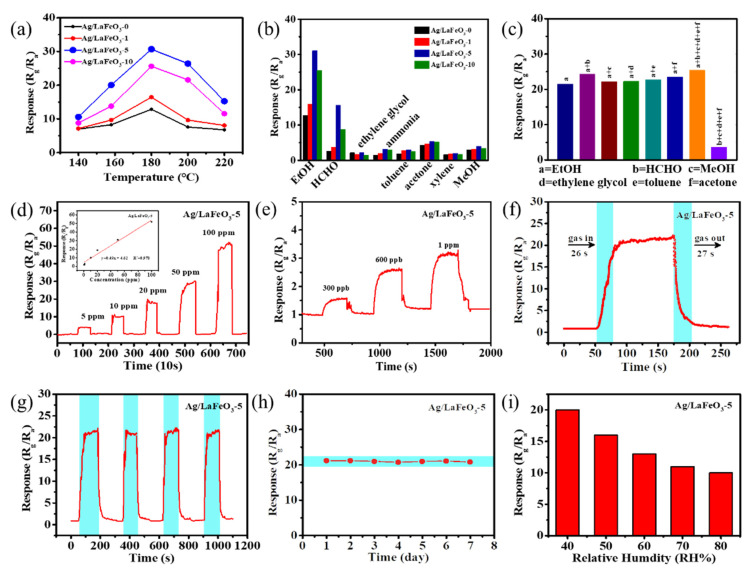
(**a**) The response of Ag/LaFeO_3_-0--Ag/LaFeO_3_-5 nanomaterials to 50 ppm ethanol in the range of 140–220 °C; (**b**) selectivity of the four Ag/LaFeO_3_ sensors for different gases at 180 °C (concentrations of 50 ppm for all gas); (**c**) sensor response values for Ag/LaFeO_3_-5 at 180 °C for a mixture of vapors (20 ppm concentration of these gases); (**d**) response curves of the Ag/LaFeO_3_-5 sensor for EtOH vapors ranging from 5 ppm to 100 ppm concentration, the inset exhibits a direct linearity correlation between the sensing response values and EtOH vapor levels; (**e**) dynamic response curve of Ag/LaFeO_3_-5-based sensors under a low concentration of EtOH exposed at 180 °C; (**f**) sensing curves and (**g**) reproducibility of the Ag/LaFeO_3_-5 sensor for 20 ppm EtOH vapors; (**h**) the long-term stability of sensitive materials; (**i**) response of Ag/LaFeO_3_-5-based sensors to 20 ppm EtOH under different RH%.

The operating temperature is correlated with the carrier concentration and surface reaction activation energy of gas-sensitive materials, which is one of the major factors that affect the gas-sensitive performance. To explore the best working temperature of the four Ag/LaFeO_3_ nanosheets, a series of tests were conducted on ethanol at 50 ppm over a temperature range of 140–220 °C (Figure 5a). Apparently, the response value first tended to increase and then decrease as the operating temperature increased. The initial growth in response values was associated with the activation of the gas molecules/sensing material and the acceleration of electron conduction. With a further increase in temperature, the adsorption rate fell far short of the desorption rate and fewer gas molecules were trapped on the interface of the material, which led to smaller response values. At the same time, the sensing response of all Ag/LaFeO_3_ samples showed a similar trend, with their sensitivity reaching a maximum at 180 °C, respectively. The modification of Ag increased the sensitivity of the LaFeO_3_ sensor to ethanol, presenting a trend of an increasing and then decreasing response as the Ag content increased. In this case, the number of surface defects (i.e., active sites) did not always increase with the amount of Ag modification [37]. At 5% Ag modification, the concentration of surface defects attained a maximum value, where the corresponding sensor sensitivity was optimal. Sensing performance suffered at a higher Ag-modified amount caused by the aggregation of Ag nanoparticles and the reduction in catalytic active sites [39]. Accordingly, 180 °C was considered the best operating temperature for further gas sensitivity testing.

The sensitivity of the four Ag/LaFeO_3_ sensors was investigated. Seven interfering gases, including formaldehyde, ethylene glycol, ammonia, toluene, acetone, xylene and methanol were selected as the interfering gases. As the image shows, the results confirmed that the four sensors showed some similarity in selectivity, and the response values of these sensors were higher for ethanol than for other gases (Figure 5b). Therefore, it was tentatively concluded that the gas sensors prepared by Ag-modified ultrathin LaFeO_3_ nanosheets had a significant selectivity to ethanol. Moreover, the Ag/LaFeO_3_-5-based gas sensors showed a better selectivity for ethanol gas than the other three sensors. Hence, Ag/LaFeO_3_-5 nanosheets were considered a promising method for the selective detection of ethanol gas in complex environments. It was also vital to selectively and accurately detect the presence of ethanol from the gas mixture. When the Ag/LaFeO_3_-5 sensors were exposed to an artificial atmosphere with an ethanol mixture, the sensing signal remained close to the response when ethanol vapor alone was present (Figure 5c). Therefore, the Ag/LaFeO_3_-5 sensors demonstrated a good interference immunity for the detection of ethanol.

Figure 5d demonstrates that the Ag/LaFeO_3_-5 sensor exhibited a dynamic response in ethanol from 5 to 100 ppm. The response values of the sensors showed a step-growth as the ethanol concentration in the chamber increased from 5 ppm to 100 ppm. In addition, the gas sensor was tested for ethanol vapor atmospheres of 5, 10, 20, 50 and 100 ppm, corresponding to response values of 3.9, 9.9, 20.9, 30.5 and 50.6, respectively. Furthermore, the inset c clearly shows that the sensor exhibited a good linearity in the range of 5 to 100 ppm for ethanol vapor concentrations. The fitted curve was available as a function y = 0.49x + 0.46, where the value of R^2^ was 0.98. Thus, the results showed that none of the Ag/LaFeO_3_-5-based sensors saturated at ethanol gas concentrations below 100 ppm. Considering the performance of sensitive materials to test for low concentrations of ethanol and to obtain detection limits, the gas-sensing potential of these gas sensors was measured over the concentration ranges of 300~1000 ppb of ethanol gas (Figure 5e). Significantly responsive to low gas concentrations (R_a_/R_g_ = 1.6), the sensor showed considerable promise for the capture of trace amounts of ethanol.

Response and recovery times perform a critical role in the monitoring ability of gas sensors. Generally, the faster the response/recovery time, the better the gas sensor performance will be. A graph of the recovery curve of the response to 20 ppm ethanol vapor based on the Ag/LaFeO_3_-5 sensors is given in Figure 5f. The response/recovery time of the ethanol gas sensors was 26/27 s, which could meet the demand for gas detection in real life. Repeatability and stability are important considerations in routine applications. Figure 5g shows four dynamic cycles of response and recovery for 20 ppm ethanol. Noticeably, the response and recovery resistance of the gas sensor did not vary visibly after four cycles of measurement, which demonstrated a good repeatability. Long-term tests on these gas sensors have revealed that their performance could be maintained over one week with response values of around 95% (Figure 5h). Therefore, the Ag/LaFeO_3_-5 sensor had a good repeatability and stability. Figure 5i demonstrates the impact of humidity on the response of the alcohol sensor, and it was found that the gas response value tended to decrease as the humidity in the ethanol atmosphere increased.

A comparison of the performance of the Ag/LaFeO_3_-5 sensor in this work with other ethanol-based sensors previously reported is summarized in Table 1. Most LaFeO_3_-based gas sensors responded to C_2_H_5_OH at high temperatures. However, the Ag-LaFeO_3_-5 sensors showed a much warmer gas response at lower working temperatures. Consequently, taking the gas response and the operating temperature into account, Ag-LaFeO_3_-5-based sensors have a relatively advanced commercial application compared to other P-type sensors reported in the literature.

### 3.3. Gas-Sensing Mechanism

**Figure 6:**
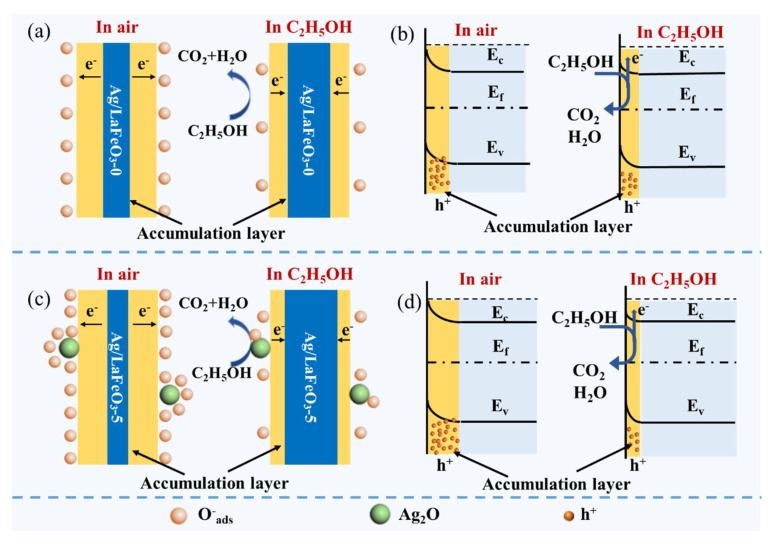
Schematic diagrams of the mechanism for the C_2_H_5_OH sensing on the (**a**,**b**) Ag/LaFeO_3_-0 nanosheets; (**c**,**d**) Ag/LaFeO_3_-5 nanosheets.

As a classic p-type semiconductor, the holes are the main carriers of LaFeO_3_. Its gas-sensitive mechanism focuses on gas adsorption. As illustrated in Figure 6a, when the gas sensor is exposed to air, oxygen molecules are potentially attracted to the surface of the LaFeO_3_ material, thus leading to the formation of adsorbed oxygen anion species through the conductivity of trapping free electrons. This step creates an increase in the carrier, which means that a high potential barrier and a deep cavity buildup layer are formed, as described as follows [10,11,44]:

O_2_ (gas) ↔ O_2_ (ads) (1)

O_2_ (ads) + e^−^ ↔ O_2−_ (ads) (T < 100 °C) (2)

O_2−_ (ads) + 2e^−^ ↔ 2O^−^ (ads) (100 °C < T < 300 °C) (3)

O^−^ (ads) + e^−^ → O^2−^ (ads) (T > 300 °C) (4)

As mentioned above, the optimum operating temperature for the Ag/LaFeO_3_-based sensor was 180 °C. The oxygen anion attached to the Ag/LaFeO_3_ material surface was primarily present in the form of O^−^. When ethanol vapor was injected into the chamber to act as a reducing gas, the oxygen species reacted with the ethanol gas while the electrons captured by the oxygen anion were freed, which returned to the conductive band of the Ag/LaFeO_3_. The electrons were annihilated by holes, resulting in an extension in the resistance of the sensitive material (Figure 6b),as described in Equations (5)–(6) [11,45]:

CH_3_CH_2_OH + 6O^−^ → 2CO_2_ + 3H_2_O + 6e^−^ (5)

h^+^ + e^−^ = null (6)

As shown in Figure 6b,c, when the Ag/LaFeO_3_-5 sensors were exposed to air, a larger number of oxygen molecules were attached to the surface of the material, which allowed more free electron binding in the conduction band to form O^−^, and the hole accumulation layer expanded compared to the pure sample. Equally, when the Ag/LaFeO_3_-5 material surface was exposed to ethanol gas, the O^−^ interacted with further ethanol molecules trapped on the material surface, releasing additional collected electrons into the conduction band, greatly narrowing the hole accumulation layer and substantially increasing the resistance [29,46]. In addition, this outstanding sensing performance could be credited to the chemical and electronic sensitivity of Ag nanoparticles [39,47]. As well as being an efficient catalyst for the oxidation of ethanol, the Ag NPs distributed on the LaFeO_3_ surface are also potent catalysts for the adsorption–desorption reaction of oxygen [38,48]. To investigate the gas-sensing mechanism in depth, some studies have been performed based on the density flooding theory (DFT) with theoretical calculations explaining that the selectivity of LaFeO_3_-based sensors for ethanol can probably be attributed to the much higher adsorption energy of ethanol gas on the sensor surface than other gases [13,49]. It was also suggested that the lowest unoccupied molecular orbital (LUMO) energy values of various volatile organic compounds could reflect the gas-sensing sensitivity [50]. The high adsorption energy of ethanol on the LaFeO_3_-based sensor surface combined with the low LUMO energy value of C_2_H_5_OH may help to explain the selective detection of ethanol in this study. As a result, the Ag/LaFeO_3_-5 sensor offered a higher ethanol gas response compared with pure LaFeO_3_. Nevertheless, more efforts are still expected to further clarify the details of the current mechanism of the Ag/LaFeO_3_ system owing to the complexity of the gas-sensing mechanism.

## 4. Conclusions

In conclusion, the ultrathin two-dimensional LaFeO_3_ nanosheets were successfully prepared using the GO template method. The XRD and SEM results showed that the LaFeO_3_ nanosheets obtained by calcination at 700 °C were well crystalline and had an ultrathin two-dimensional porous nanosheet morphology. The Ag-modified ultra-thin porous LaFeO_3_ nanosheets were investigated, and the LaFeO_3_ nanosheets calcined at 700 °C were surface modified with Ag to obtain gas-sensitive materials with a higher gas-sensitive response to ethanol. By investigating the effect of Ag modification on the gas-sensitive performance, the Ag/LaFeO_3_-5 nanosheets were shown to achieve a response of 30.5 at the optimum working temperature of 180 °C towards 50 ppm ethanol, which was about 2.4 times higher than that of the gas-sensitive material without Ag modification (Ag/LaFeO_3_-0). Therefore, Ag-modified LaFeO_3_ has considerable prospects for the potential development of simple and economical alcohol gas sensors for practical applications.

## Figures and Tables

**Table 1:** Comparison of sensor-based ethanol gas detection characteristics with different detection materials.

Materials	T (°C)	Concentration (ppm)	Response (R_g_/R_a_)	T_res_/T_rec_ (s)	Ref.
rGO/CoTiO_3_	195	50	9	2/5	[40]
rGO/Co_3_O_4_	200	100	21		[41]
Ba-doped LaFeO_3_	210	100	31.3	40/42	[11]
NiO@LaFeO_3_	240	10	14.7	2/9	[42]
LaFeO_3_	300	143	14.5	23/29	[43]
Ag/LaFeO_3_-5	180	20	21	26/27	This work

## Data Availability

Data is contained within the article or Supplementary Materials.

## Supplementary Materials

The following supporting information can be downloaded at: https://www.mdpi.com/article/10.3390/nano12101768/s1, Figure S1: The schematic structure of the gas sensor, Figure S2: (a) TG curves of LaFeO_3_/GO, (b) Raman spectra of the LaFeO_3_ samples with different calcination temperatures, Figure S3: Response of LaFeO_3_ samples toward 50 ppm of EtOH at operation temperature ranging from 140 to 220 °C. Table S1: Fitting results of O 1s XPS spectra of Ag/LaFeO_3_-0 and Ag/LaFeO_3_-5.
