# Supplementary material for: Ag-Modified Porous Perovskite-Type LaFeO3 for Efficient Ethanol Detection
Source: Nanomaterials (Basel). 2022 May 22;12(10):1768. doi: 10.3390/nano12101768 (PMC9143232; doi:10.3390/nano12101768)
Supplement: Supplementary file 1 [file nanomaterials-12-01768-s001.zip › nanomaterials-1731579-supplementary.pdf]

nanomaterials

Supporting Information

Ag-Modified Porous Perovskite-Type $LaFeO_3$ for Efficient Ethanol Detection

Jiejie Yu [1,†], Cong Wang [1,†], Quan Yuan [1], Xin Yu [1], Ding Wang [1,*], Yang Chen [2,3,*]

1 School of Materials and Chemistry, University of Shanghai for Science & Technology, Shanghai 200093, China; m15370026136@163.com (J.Y.); wangcong_0311@163.com (C.W.); yquan0327@163.com (Q.Y.); yuxin156625861@163.com (X.Y.)
2 NEST Lab, Department of Physics, Department of Chemistry, College of Sciences, Shanghai University, Shanghai 200444, China
3 Shanghai Yaolu Instrument & Equipment Co., Ltd., Shanghai 200444, China
* Correspondence: wangding@usst.edu.cn (D.W.); shucy@shu.edu.cn (Y.C.)
† These authors contributed equally to this work.

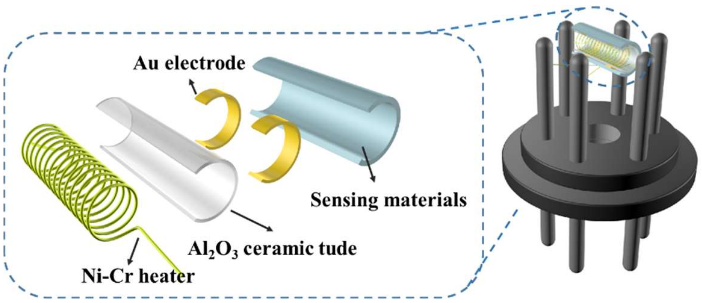

Figure S1. The schematic structure of the gas sensor.

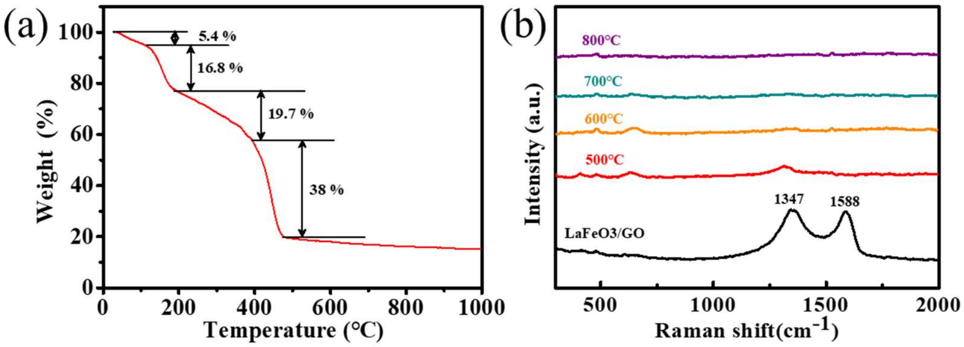

Figure S2. (a) TG curves of $LaFeO_3$/GO, (**b**) Raman spectra of the $LaFeO_3$ samples with different calcination temperatures.

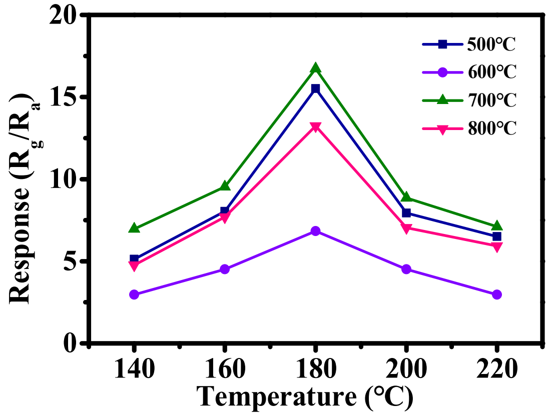

Figure S3. Response of $LaFeO_3$ samples toward 50 ppm of EtOH at operation temperature ranging from 140 to 220 °C.

Table S1. Fitting results of O 1s XPS spectra of Ag-$LaFeO_3$-0 and Ag-$LaFeO_3$-5.

Samples		O_{lat}	O_{def}	O_{abs}
Ag-$LaFeO_3$-0	Binding enengy (eV)	528.7	531.2	532.5
	Relative percentage (%)	46.71	46.55	6.74
Ag-$LaFeO_3$-5	Binding enengy (eV)	528.6	531.2	532.4
	Relative percentage (%)	46.25	44.28	9.47
